# Using a System Identification Approach to Investigate Subtask Control during Human Locomotion

**DOI:** 10.3389/fncom.2016.00146

**Published:** 2017-01-11

**Authors:** David Logan, Tim Kiemel, John J. Jeka

**Affiliations:** ^1^Department of Kinesiology, University of MarylandCollege Park, MD, USA; ^2^Department of Kinesiology, Temple UniversityPhiladelphia, PA, USA; ^3^Department of Bioengineering, Temple UniversityPhiladelphia, PA, USA

**Keywords:** human locomotion, sensorimotor control, harmonic transfer functions, phase-dependent impulse response functions, subtask control

## Abstract

Here we apply a control theoretic view of movement to the behavior of human locomotion with the goal of using perturbations to learn about subtask control. Controlling one's speed and maintaining upright posture are two critical subtasks, or underlying functions, of human locomotion. How the nervous system simultaneously controls these two subtasks was investigated in this study. Continuous visual and mechanical perturbations were applied concurrently to subjects (*n* = 20) as probes to investigate these two subtasks during treadmill walking. Novel application of harmonic transfer function (HTF) analysis to human motor behavior was used, and these HTFs were converted to the time-domain based representation of phase-dependent impulse response functions (ϕIRFs). These ϕIRFs were used to identify the mapping from perturbation inputs to kinematic and electromyographic (EMG) outputs throughout the phases of the gait cycle. Mechanical perturbations caused an initial, passive change in trunk orientation and, at some phases of stimulus presentation, a corrective trunk EMG and orientation response. Visual perturbations elicited a trunk EMG response prior to a trunk orientation response, which was subsequently followed by an anterior-posterior displacement response. This finding supports the notion that there is a temporal hierarchy of functional subtasks during locomotion in which the control of upper-body posture precedes other subtasks. Moreover, the novel analysis we apply has the potential to probe a broad range of rhythmic behaviors to better understand their neural control.

## Introduction

Treadmill walking is very useful to study the neural control of locomotion as it constrains locomotive behavior, at a minimum, to two requirements. First, treadmill walking requires subjects adjust their speed so that they do not fall off the front or back of the treadmill. Second, as in any walking task unaided by weight support, subjects must maintain orientation relative to vertical and not allow the proportionally massive trunk to topple over the legs. What is less clear is how the nervous system simultaneously adjusts speed for maintaining position and trunk orientation for upright posture, which is the focus of this study.

Here we use visual and mechanical perturbations, as both have been used separately to successfully learn about subtasks during walking. Changes in virtual visual scene motion have been previously used to alter speed (Konczak, 1994), trunk orientation to vertical (Logan et al., 2010), stride length (Prokop et al., 1997), translation of the body on the treadmill (Warren et al., 1996; Logan et al., 2010), speed of the walk-run transition (Mohler et al., 2007) and its kinematic/energetic features (Guerin and Bardy, 2008).

Mechanical perturbations during walking have also been used to investigate many subtasks of walking. An early investigation by Nashner made use of support surface perturbations to show that stabilizing muscle activations during walking mimicked those occurring during standing posture (Nashner, 1980), reflecting postural control within locomotion. Further investigation into postural control during walking revealed that subjects will first stabilize posture prior to performing an additional, planned lever pulling task (Nashner and Forssberg, 1986). Mechanical perturbations have also been used to study the subtask of obstacle avoidance/ accommodation during walking, and have revealed an elevating or lowering strategy (Eng et al., 1994) or mixture of the two (Forner Cordero et al., 2003) depending on phase of the gait cycle. More recently, Ahn and Hogan (2012) used torque perturbations at the ankle and found that the gait period will entrain to the perturbation when advantageous for propulsion, supporting a neuro-mechanical oscillator for propulsion control. The authors interpreted these findings as a separation in control of low level propulsion and higher level “episodic supervisory control of a semi-autonomous periphery” when needed for cases, such as irregular footholds or obstacle avoidance, compatible with a subtask-dependent control scheme. In sum, visual and mechanical perturbations have been previously used in isolation to provide insight into human walking control.

**Figure 1:**
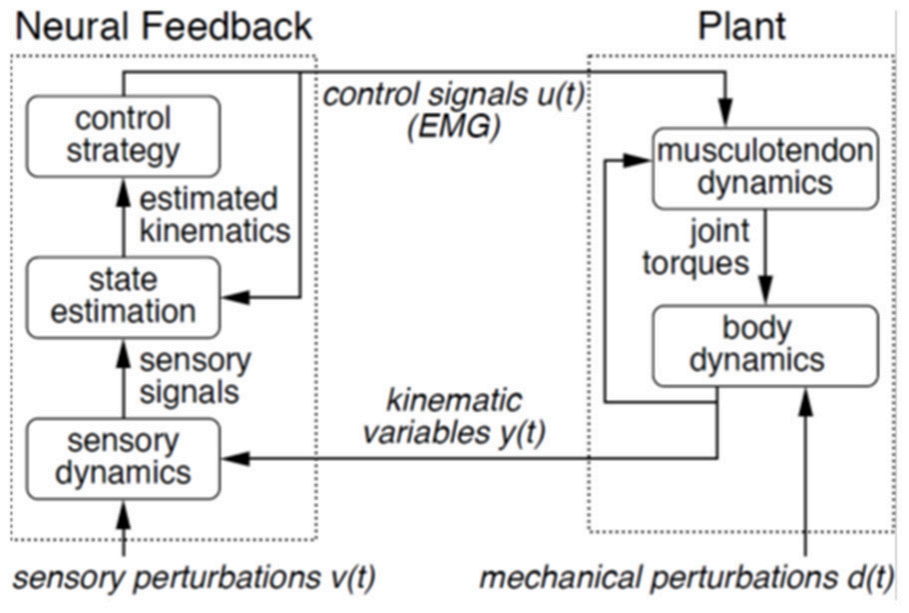
**Control theoretic view of motor behavior**. In this model, motor behavior consists of two components: musculoskeletal plant and neural feedback. The plant is composed of joint torques produced by musculotendon dynamics and ensuing body dynamics, with muscle activity as precursor. Feedback consists of those sensory signals arising from sensory systems, which update the neural controller based on orientation and movements of the body. Positions and velocities are estimated (state estimation), and appropriate motor commands (control strategy) are specified in the feedback portion of the control loop.

Here we used simultaneous virtual scene motion and distributed pulling at the back of the trunk to probe the control of treadmill walking. Using the control theoretic view of movement shown in Figure 1 (Kiemel et al., 2008, 2011; Logan et al., 2010), we sought to perturb treadmill walking at distinct points in the control loop to investigate whether the nervous system changes the priority of different subtasks. Our assumption is that scene motion in an immersive virtual environment perturbs the sensorimotor feedback portion of the control loop and a motor attached to the upper trunk through a spring mechanically perturbs the musculoskeletal plant (see Figure 1). The mechanical perturbation first moves the body, which then elicits active (neurally-driven) electromyographic (EMG) responses. In contrast, a visual perturbation first elicits muscle activation, which then moves the body. Using these perturbations simultaneously in this investigation is a step toward understanding both the control problem (musculoskeletal plant) that the nervous system faces and its solution (neural feedback) during bipedal locomotion.

To do so we used small, continuous perturbations, which are considered probes of the control structure and are less likely to change the control structure (e.g., increased effective stiffness). We sought to probe walking with perturbations that yield small, significant deviations of response variables (kinematics, EMG) from mean behavior for insight into the closed-loop control system. Perturbations across gait cycle phases were used as the effects of visual and mechanical perturbations during walking will, in general, depend on the phase of the gait cycle at which they are applied (Nashner, 1980; Nashner and Forssberg, 1986; Eng et al., 1994; Forner Cordero et al., 2003; Logan et al., 2014). The effects of continuous perturbations on response variables were characterized with a novel application of phase-dependent impulse response functions (ϕIRFs, where we use “ϕ” to denote phrase-dependence) to the study of human walking (Kiemel et al., 2016, pre-print available at http://arxiv.org/abs/1607.01746). For a linear time periodic (LTP) system with input *u*(*t*) and output *y*(*t*), a ϕIRF *h*(*t*_r_, *t*_*s*_) describes the response at time *t*_r_ to an impulse applied at time *t*_*s*_ (Möllerstedt and Bernhardsson, 2000). For a nonlinear system with a stable limit cycle, a ϕIRF approximates its response to any small transient perturbation:

(1) $$y(t_{\text{r}})=y_{0}(t_{\text{r}})+\underset{-\infty }{\int^{t_{\text{r}}}}h(t_{\text{r}},t_{s})u(t_{s})dt_{s},$$

where *y*_0_(*t*_r_) is the unperturbed periodic output.

The ϕIRF of an LTP system can be computed directly in the time domain using ensemble methods for general linear time-varying systems (Soechting et al., 1981; Lacquaniti et al., 1982; MacNeil et al., 1992). Ludvig and Perreault (2012) noted that these methods may require many experimental trials (realizations) and proposed a more efficient method that is applicable for an LTP system in which ϕIRF responses decay quickly relative to the system's cycle period. The ϕIRF can be computed efficiently without this constraint by first computing a harmonic transfer function (HTF) in the frequency domain (Wereley and Hall, 1990) and then converting the HTF to a ϕIRF in the time domain (Möllerstedt and Bernhardsson, 2000). However, methods used to compute the ϕIRF of an LTP system are not necessarily valid for limit-cycle systems, because perturbations can reset the phase of the oscillator, violating the assumption of periodicity. Much of the theory for LTP systems assumes that a transient perturbation produces a transient response (Sandberg et al., 2005), which is not true when the perturbation resets phase. The novelty of the method used in this study is that it accounts for phase resetting and, thus, can be applied to walking. Our method is a modification of the HTF-to-ϕIRF method for LTP systems and retains its advantage of experimental efficiency.

**Figure 2:**
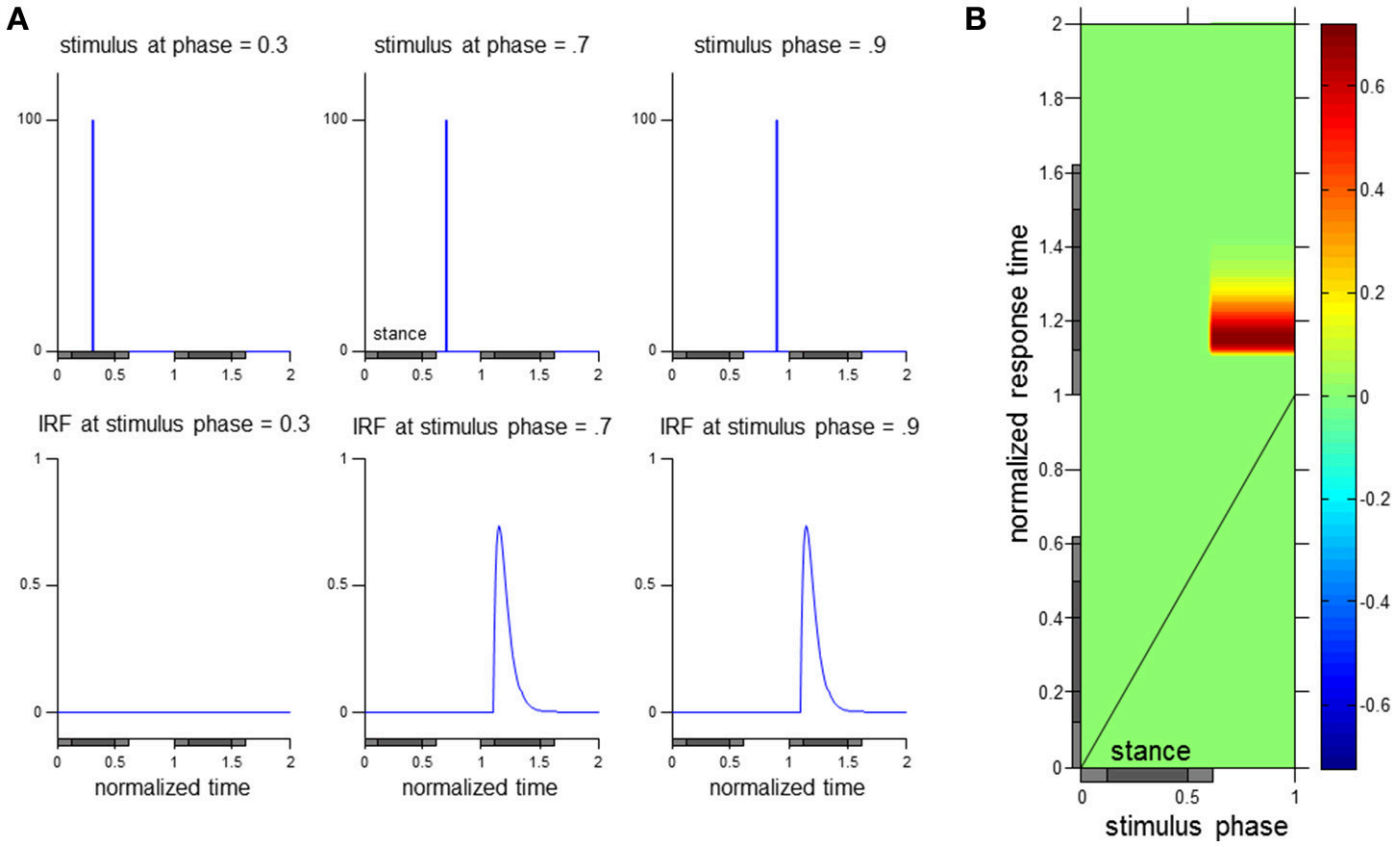
**Visualization of the ϕIRF**. Hypothetical responses to discrete perturbations applied at three stimulus phases and their corresponding impulse response function (IRF) are presented in **(A)**. A transfer of these discrete perturbation responses to a ϕIRF visualization in **(B)** allows observation of the input-output relationship across stimulus phase and normalized response time (see Methods for details on computation of the ϕIRF using continuous perturbations). As in the experimental data presented in this manuscript, normalized time in this hypothetical case is in gait cycle units. The gray horizontal bars below indicate stance phase with times of double support indicated with a lighter shade.

As seen in Figure 2, presenting the data as the ϕIRF allows a characterization of the input perturbation and output response variable throughout the phases of the gait cycle with respect to stimulus phase and normalized response time. Stimuli and impulse response functions of hypothetical walking data at three stimulus phases are observed in Figure 2A with corresponding visualization as a ϕIRF in Figure 2B. The ϕIRF in Figure 2B would quickly tell us in a single picture that perturbations occurring solely during swing phase yield responses in the stance phase of the following gait cycle. A ϕIRF describes the response to a small brief discrete perturbation at any phase of the gait cycle. However, it is methodologically inefficient to experimentally use discrete perturbations to determine the ϕIRF (as in Logan et al., 2014). Instead, responses to continuous perturbations are analyzed in the frequency domain and then converted to the time domain to compute the ϕIRF (see Methods and Kiemel et al., 2016).

Working within the theoretical framework shown in Figure 1, mechanical and sensory perturbations have been successfully applied to non-parametrically identify both the musculoskeletal plant (Kiemel et al., 2008) and the sensorimotor feedback (Kiemel et al., 2011) portions of the control loop during standing postural control. Here we attempt a similar identification scheme aimed at walking while simultaneously probing subtask control. Supported by the finding that postural corrections are initiated prior to performance of an additional, mechanically destabilizing task (Nashner and Forssberg, 1986), we hypothesized that both perturbations would elicit a control strategy that prioritized control of trunk orientation for staying upright over adjustments in speed to maintain position on the treadmill.

## Materials and methods

### Subjects

Twenty healthy subjects [8 males and 12 females, between 19 and 30 years. of age, 67.9 ± 12.9 kg (mean ± SD)] participated in this study. All subjects were self-reported to have normal (or corrected to normal) vision. The studies conformed to the Declaration of Helsinki, and all participants provided informed, written consent to the experimental procedures detailed in this manuscript. These experimental procedures and consent process were approved by the Institutional Review Board of the University of Maryland, College Park.

### Apparatus

#### Virtual reality environment

Subjects walked at 5 km h^−1^ on a treadmill (Cybex Trotter 900T, Cybex International, Inc., USA) surrounded by three screens (width, 3.05 m; height, 2.44 m; Fakespace, USA), one in front of the subject and one on either side. Subjects wore goggles with the top shield occluded to prevent them from seeing motion capture cameras mounted above the screen in front of them. Visual displays were rear projected to the screens at a frame rate of 60 Hz by JVC projectors (model DLA-M15U; Victor Company of Japan). CaveLib software (Mechdyne, USA) was used to generate a virtual moving visual scene consisting of three walls attached at right angles that coincide with the screens when the visual scene is not moving. Each wall consisted of 500 non-overlapping white small triangles (3.4 × 3.4 × 3.0 cm) with random positions and orientations on a black background. To reduce aliasing effects in the fovea region, no triangles were displayed on the front wall within a 30-cm-radius circular region directly in front of the participant's eyes. The display on each screen was varied in time to simulate rotation of the visual scene about the medial-lateral axis located at the subject's ankle height at 1 m from the screen, assuming a fixed perspective point at the participant's eye height 1 m from the screen. The signals specifying scene-rotation angle were created offline (Matlab, Mathworks, USA) and were generated via Labview (National Instruments, USA) on a desktop computer (Precision T5500, Dell, USA).

#### Mechanical perturbation

**Figure 3:**
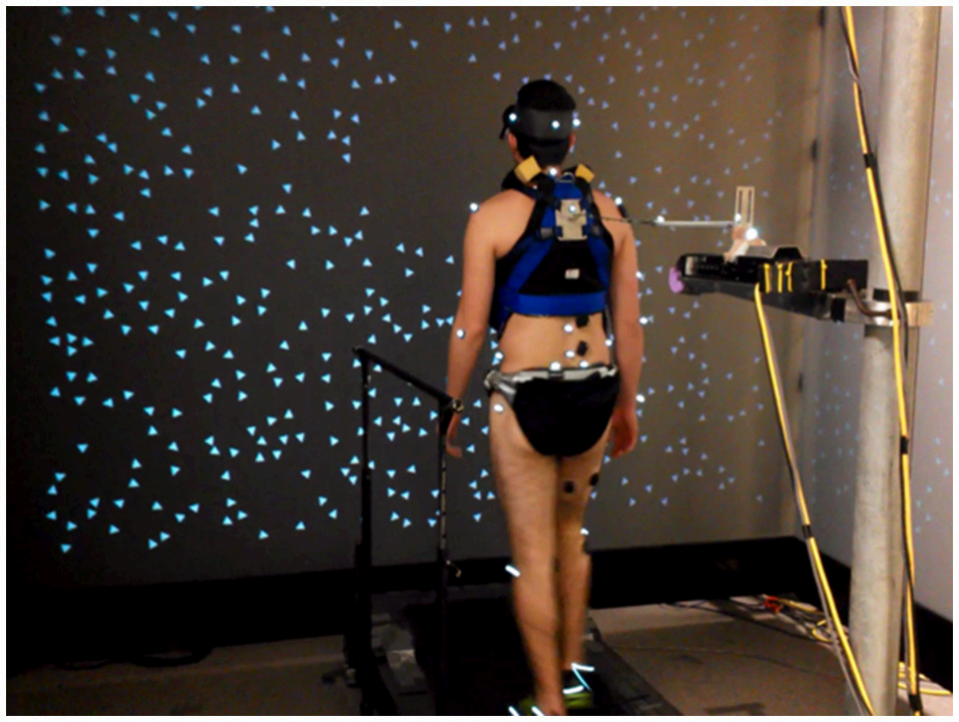
**Experimental setup**. Subjects walked on a treadmill located within a three panel virtual “cave” providing rotating visual scene motion in the sagittal plane. Subjects were also attached to a motor through a spring and rigid cable in series.

As seen in Figure 3, a weak continuous mechanical perturbation was applied to the subject from behind as a spring with one end attached to a modified trunk harness worn by the subject and the other end attached to a linear motor (LX80L; Parker Hannifin Corporation). The spring was attached in series with a 45.7 cm rigid cable fixed to the back of the harness. The harness was adjusted for each subject so that the point of attachment was at mid-scapula height centered on the midline of the upper trunk. The actual displacement of the motor in the anterior posterior (A-P) direction, as indicated by a VICON reflective marker on the motor, was used as the mechanical perturbation signal. The force on the body was *F*(*t*) = *k*(*u*(*t*) − *y*(*t*) − *u*_0_), where *k* is the spring constant, *u*(*t*) is the perturbation signal, *y*(*t*) is the A-P position of the point on the body at which the perturbation is applied, and *u*_0_ is a constant such that *F*(*t*) < 0 (force in the backward direction) throughout each trial. We used a weak spring (*k* = 0.0175 N/mm) so that the effect of the mechanical perturbation on gait kinematics and EMG signals would be small. Since *k* is small, the ϕIRF for our mechanical perturbation is approximately equal to *k* times the ϕIRF that would be measured if, instead of specifying motor position, we would have specified the force applied to the body.

#### Perturbation signals

Both visual and motor signals were filtered white noise signals. For each trial of each subject and each perturbation type, a different seed was used to generate a white noise signal using a random number generator. To create a signal specifying the angle of the visual scene, white noise with a one-sided spectral density of 150 deg^2^/Hz was filtered using a first-order low-pass filter with a cutoff frequency of 0.02 Hz and a second-order Butterworth low-pass filter with a cutoff frequency of 5 Hz. Across subjects, these visual signals had an average root mean square (RMS) value of 2.13 deg. In our analysis (described below), visual-scene angular velocity was used as the perturbation signal. The RMS velocity of visual signals, averaged across subjects, was 3.62 deg/s. A positive/negative signal corresponded to a forward rotation into the screen/backward rotation toward the subject.

To create a signal specifying the position of the motor, white noise with a one-sided spectral density of 1.1 cm^2^/Hz was filtered using an eighth-order Butterworth low-pass filter with a cutoff frequency of 4 Hz. Across subjects, these driving signals had an average RMS position of 1.30 cm and RMS velocity of 19.40 cm/s. These parameters were used for the motor signal as a balance between ensuring a flat power spectrum up to highest frequency possible and staying within traveling distance and velocity limits of the motor. Visual display generation, motor motion, and data collection software were synchronized via an external trigger. Furthermore, EMG data were synchronized in time with rest of the experimental setup by correcting for a 48 ms group delay occurring when analog output is used by TRIGNO (DELSYS, USA) EMG system.

#### Kinematics

Body kinematics were measured using a 10 camera VICON-MX motion analysis system (VICON, Inc, Oxford, UK). Reflective markers (diameter, 1.4 cm) were placed on the right and left sides of the body at external landmarks corresponding to: base of the 5th metatarsal, posterior calcaneus (heel), lateral malleolus (ankle), lateral femoral condyle (knee), greater trochanter (hip), anterior superior iliac spine (ASIS), posterior superior iliac spine (PSIS), iliac crest, superior acromion process (shoulder), mastoid process (head) and frontal eminence (head). Additionally, markers were placed at the medio-lateral center of the back of the head and the midline of the spine at the level of C6, T10, and L1 vertebrae. All markers were attached at the skin of these bony prominences except those placed on the shoe at the 5th metatarsal and heel. All kinematic data were collected at 120 Hz.

Our analysis focuses on the trunk segment in the sagittal plane as well as whole-body displacements in the A-P direction. Trunk orientation relative to the vertical in the sagittal plane was computed as the angle formed by the L1 to T1 markers. Whole-body displacement in the A-P direction was measured as the displacement of L1 in the A-P direction.

#### Muscle activity (sEMG)

Muscular activity of the right leg and trunk was measured using surface electromyographic (sEMG) recordings. Recordings of the following 16 muscles were made: tibialis anterior, gastrocnemius lateralis, gastrocnemius medialis, soleus, vastus medialis, vastus lateralis, rectus femoris, tensor fascia latae, biceps femoris, semitendinosus, gluteus maximus, gluteus medius, rectus abdominus, lumbar erector spinae, thoracic erector spinae (EST, recorded at T9), and posterior deltoid. Electrodes were positioned at the muscle belly with placement carefully chosen to minimize cross-talk (Cappellini et al., 2006). Recording sites were shaved, lightly abraded, and cleaned with isopropyl alcohol prior to electrode application. The sEMG data were recorded at 2160 Hz using the wireless TRIGNO system (DELSYS, USA). This recording system has built in bandwidth of 20–450 Hz and gain of 909 V/V. Using Matlab, these signals were high-pass filtered using a zero-lag forward-backward cascade of a 4th order Butterworth filter with a 20-Hz cutoff frequency, full-wave rectified, and then low-pass filtered with a zero-lag forward-backward cascade of a 4th order Butterworth filter with a 10-Hz cutoff frequency. Although consistent sEMG responses were observed in many muscles to the visual perturbation, we focus on an erector spinae muscle (EST) in the results presented below as consistent responses were observed solely in this muscle for both perturbations.

### Procedures

Prior to experimentation, subjects experienced a static visual display at the experimental locomotion speed. An experimenter was always behind the treadmill in close proximity to the subject to ensure safety in case of falling (never occurred). Subjects began each experimental trial by looking straight ahead at the static visual display at the experimental treadmill speed (5 km/h) for approximately 30 s to reach steady-state treadmill walking. At this point, the subject would declare if he or she was ready for the trial to begin. The experimenter then initiated data acquisition, scene motion and the motor simultaneously with variable delays on each trial to avoid start-up effects. Each trial was 250 s in duration with a rest of at least 60 s between trials. The initial and final 5 s of each 250 s signal were multiplied by increasing and decreasing ramps, respectively, to insure that the value of the signal at the beginning and end of the trial would be 0. Only the middle 240 s of each trial was analyzed. The experimental design consisted of 10 trials of visual scene and motor motion. Upon inspection of trajectories of the kinematic marker on the spring attached to the motor there were instances where the spring clearly went slack during the trial. These instances were removed from analysis, resulting in shorter trials in 13 of the 200 trials recorded across subjects.

### Data analysis

#### Phase-dependent impulse response functions

Here we describe the analysis steps used to compute (ϕIRFs). A fuller description with equations and expanded motivation can be found in Kiemel et al. (2016, pre-print available at http://arxiv.org/abs/1607.01746). Our method is based on existing theory for linear time-periodic systems (e.g., Wereley and Hall, 1990; Möllerstedt and Bernhardsson, 2000; Sandberg et al., 2005) extended for general limit-cycle systems in which perturbations can reset the phase of the oscillator. Our method assumes that the system has smooth dynamics (see Ankarali and Cowan, 2014 for a method designed for hybrid LTP systems). The goal of the analysis is to describe the effect of *u*(*t*), a visual scene velocity or motor position perturbation, on *y*(*t*), a kinematic or sEMG response variable. **The majority of results presented are full ϕ**IRF**s, and are calculated in step 6**. Computing the full ϕIRF consists of six steps:

1. **Approximate phase**. First we compute heel-strike times *t*_*k*_(*k* = 1, …, *K*) for a reference leg. Then we compute $\bar{T}$, the mean of the stride times *t*_*k* + 1_ − *t*_*k*_(*k* = 1, …, *K* − 1), and compute the estimated gait frequency as $f_{0}=1/\bar{T}$. Next we define a discontinuous approximation of phase as θ_d_(*t*) = *k* + *f*_0_(*t* − *t*_*k*_) for *t*_*k*_ ≤ *t* < *t*_*k* + 1_. Approximate phase θ_d_(*t*) is designed to be causal, that is, to only depend on data up to and including time *t*. To obtain a continuously-differentiable causal approximation of phase, θ(*t*), we apply a second-order low-pass filter to θ_d_(*t*):
  $$\ddot{\theta }(t)+2d(\dot{\theta }(t)-f_{0})+d^{2}\theta (t)=d^{2}\theta_{\text{d}}(t).$$
  Here d represents the filter rate constant for estimating phase, which was 2. Note that for strictly periodic gait, approximate phase θ(*t*) matches the usual definition of the phase of the gait cycle.
2. **Replace time with approximate phase**. Let p be the inverse of θ: *p*(θ(*t*)) = *t* and θ(*p*(ϑ)) = ϑ. Let approximate phase ϑ take the place of time *t* = *p*(ϑ) as the independent variable and compute *ũ*(ϑ) = *u*(*p*(ϑ)), *ỹ*(ϑ) = *y*(*p*(ϑ)), and $\tilde{q}\left(\vartheta \right)=\overset{\cdot }{\theta }\left(p\left(\vartheta \right)\right).$ (We use the symbol ϑ to distinguish approximate phase as an independent variable from approximate phase as a function of time.)
3. **Compute output variables for harmonic transfer function (HTF) analysis**. For each ϑ, let *ỹ*_0_(ϑ) be the mean of *ỹ*(ϑ). Then compute the deviations ${ỹ}^{\left(1\right)}\left(\vartheta \right)=ỹ\left(\vartheta \right)-\;{ỹ}_{0}\left(\vartheta \right)$ and ${\tilde{q}}^{\left(1\right)}\left(\vartheta \right)=\tilde{q}\left(\vartheta \right)-f_{0}$. For kinematic response variables, derivatives of position (velocity) were calculated prior to this step with integration of impulse response functions occurring after step 6.
4. **Compute transient and phase-derivative HTFs**. To account for shifts in phase that affect all response variables, both a transient and phase-derivative HTF are computed. We compute the transient HTF from *ũ*(ϑ) to *ỹ*^(1)^(ϑ), denoted ${\tilde{H}}_{y},\;$ and the phase-derivative HTF from *ũ*(ϑ) to ${\tilde{q}}^{\left(1\right)}\left(\vartheta \right)$, denoted ${\tilde{H}}_{q}$, as follows. Let *z*(ϑ) be either *ỹ*^(1)^(ϑ) or ${\tilde{q}}^{\left(1\right)}\left(\vartheta \right)$. Compute the power spectral density (PSD) *p_ũũ_*(*f*_1_) and the double-frequency cross-spectral density (CSD) *p_ũz_*(*f*_1_, *f*_2_) (Bendat and Piersol, 2000). The double-frequency CSD describes the relationship between the input signal *ũ*(ϑ) at input frequency *f*_1_ and the output signal *z*(ϑ) at output frequency *f*_2_. The PSD and CSD are computed using Welch's method with 40-cycle Hanning windows (aligned to start at an integer value of ϑ) and 50% overlap. The k-th mode of the HTF *H*_*z*_ from *ũ*(ϑ) to *z*(ϑ) is computed as *H*_*z, k*_(*f*_1_) = *p_ũz_*(*f*_1_, *f*_1_ + *kf*_0_)/*p_ũũ_*(*f*_1_). Note that *H*_*z*_ is a function of both the mode index k and the input frequency *f*_1_.
5. **Compute transient and phase ϕIRFs**. For a (LTP) mapping from *ũ*(ϑ) to *z*(ϑ), its HTF *H*_*z*_ can be converted to its ϕIRF *h*_*z*_ by a two-dimensional inverse Fourier transform. The ϕIRF *h*_*z*_ is a function of response phase ϑ_r_ and stimulus phase ϑ_s_ and can be used to represent the LTP mapping from *ũ*(ϑ) to *z*(ϑ) as
  $$z(\vartheta_{\text{r}})=\underset{-\infty }{\int^{\vartheta_{\text{r}}}}h_{z}(\vartheta_{\text{r}},\vartheta_{\text{s}})\;\tilde{u}(\vartheta_{s})d\vartheta_{\text{s}}.$$
  Using this procedure, compute the transient ϕIRF ${\tilde{h}}_{y}$ and phase-derivative ϕIRF ${\tilde{h}}_{q}$ from ${\tilde{H}}_{y}$ and ${\tilde{H}}_{q}$, respectively. Then compute the phase ϕIRF by integrating the phase-derivative ϕIRF:
  $$h_{\theta }(\vartheta_{\text{r}},\vartheta_{\text{s}})=\underset{\vartheta_{s}}{\int^{\vartheta_{r}}}{\tilde{h}}_{q}(\vartheta ,\vartheta_{\text{s}})d\vartheta .$$
6. **Compute ϕIRF**. Up to now, IRFs have been functions of response phase ϑ_r_ and stimulus phase ϑ_s_. The ϕIRFs ${\tilde{h}}_{y}$ and *h*_θ_ can be combined to obtain the ϕIRF from (*u*(*t*)) to (*y*(*t*)) that is a function of response time $t_{\text{r}}=\bar{T}\vartheta_{\text{r}}$ and stimulus time $t_{\text{s}}=\bar{T}\vartheta_{\text{s}}$:
  $$h_{y}(t_{\text{r}},t_{\text{s}})=f_{0}{\tilde{h}}_{y}(t_{\text{r}}/\bar{T},t_{\text{s}}/\bar{T})+{\tilde{y}}_{0}^{\prime }(t_{\text{r}}/\bar{T})h_{\theta }(t_{\text{r}}/\bar{T},t_{\text{s}}/\bar{T}).$$

The ϕIRF *h*_*y*_(*t*_r_, *t*_s_) resulting from this procedure describes for each *t*_r_ and *t*_s_ the response measured at time *t*_r_ due to a small brief perturbation applied at time *t*_s_. Specifically, *h*_*y*_(*t*_r_, *t*_s_) is the change in *y* divided by the integral of the perturbation. It follows that *h*_*y*_(*t*_r_, *t*_s_) = 0 for *t*_r_ < *t*_s_ and $h_{y}\left(t_{\text{r}}+\bar{T},t_{\text{s}}+\bar{T}\right)=\;h_{y}\left(t_{\text{r}},t_{\text{s}}\right).$ The usefulness of the ϕIRF lies in the fact that it describes the response for any small transient perturbation *u*(*t*), as described by (Equation 1) in Introduction, where $y_{0}\left(t_{\text{r}}\right)={ỹ}_{0}\left(t_{\text{r}}/\bar{T}\right)$. We plot a ϕIRF *h*_*y*_(*t*_r_, *t*_s_) as a function of stimulus phase $t_{\text{s}}/\bar{T}$ and normalized response time $t_{\text{r}}/\bar{T}$.

**Figure 4:**
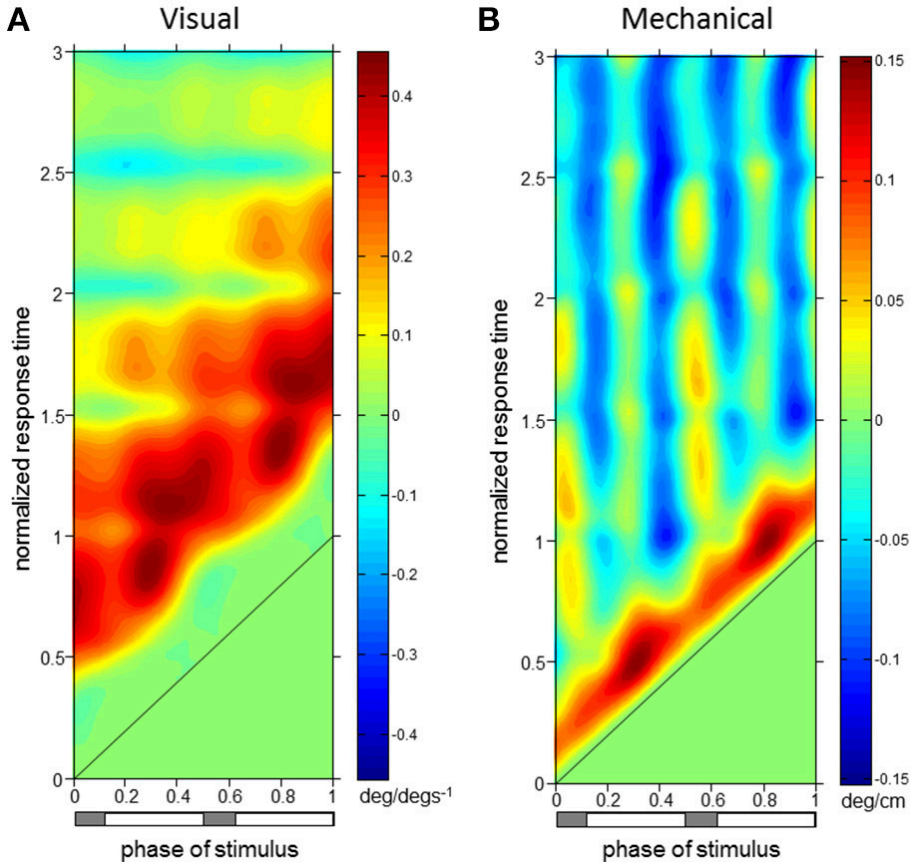
**Trunk orientation ϕIRFs. ϕIRFs from visual scene velocity (A)** and motor displacement **(B)** to trunk orientation. Intensity of colors indicate magnitude and direction at the plotted combination of stimulus phase and normalized response time. The diagonal black line is where stimulus phase is equal to the normalized response time, which indicates stimulus onset. The horizontal bar below indicates either double limb or single limb support phases in gray and white, respectively.

**Figure 5:**
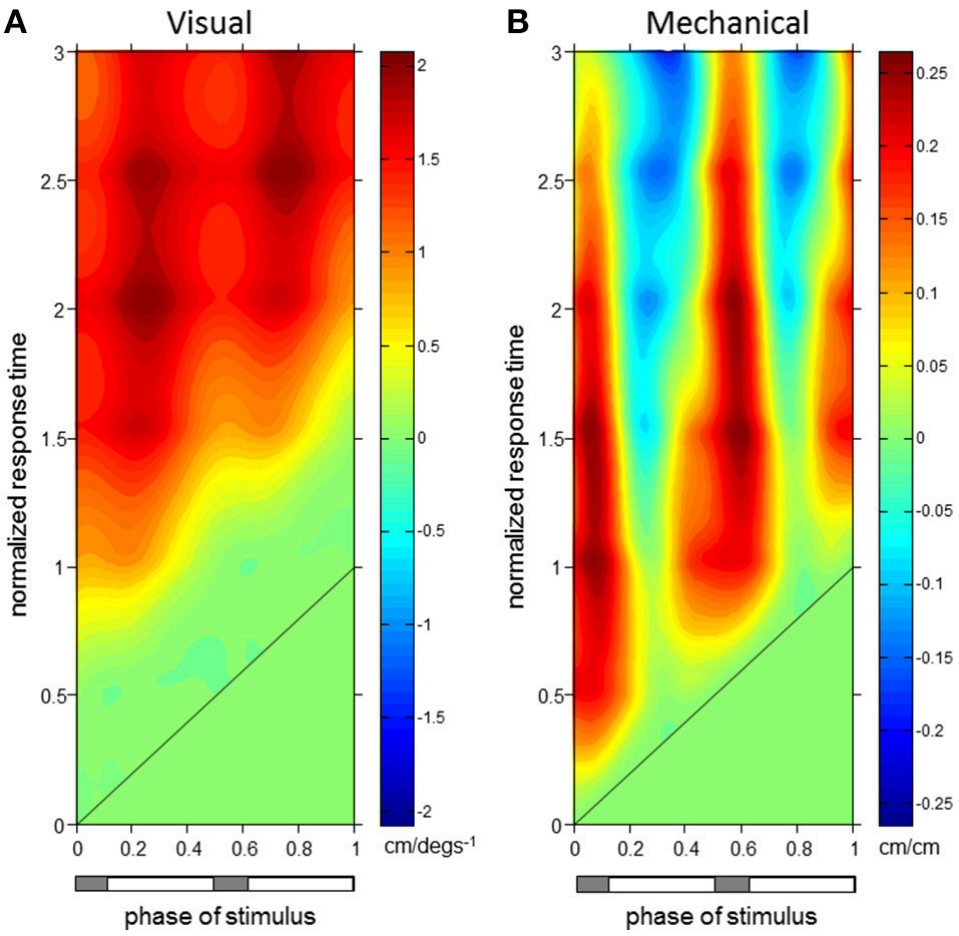
**L1 Displacement ϕIRFs. ϕIRFs from visual scene velocity (A)** and motor displacement **(B)** to L1 AP displacement. Intensity of colors indicate magnitude and direction at the plotted combination of stimulus phase and normalized response time. The diagonal black line is where stimulus phase is equal to the normalized response time, which indicates stimulus onset. The horizontal bar below indicates either double limb or single limb support phases in gray and white, respectively.

**Figure 6:**
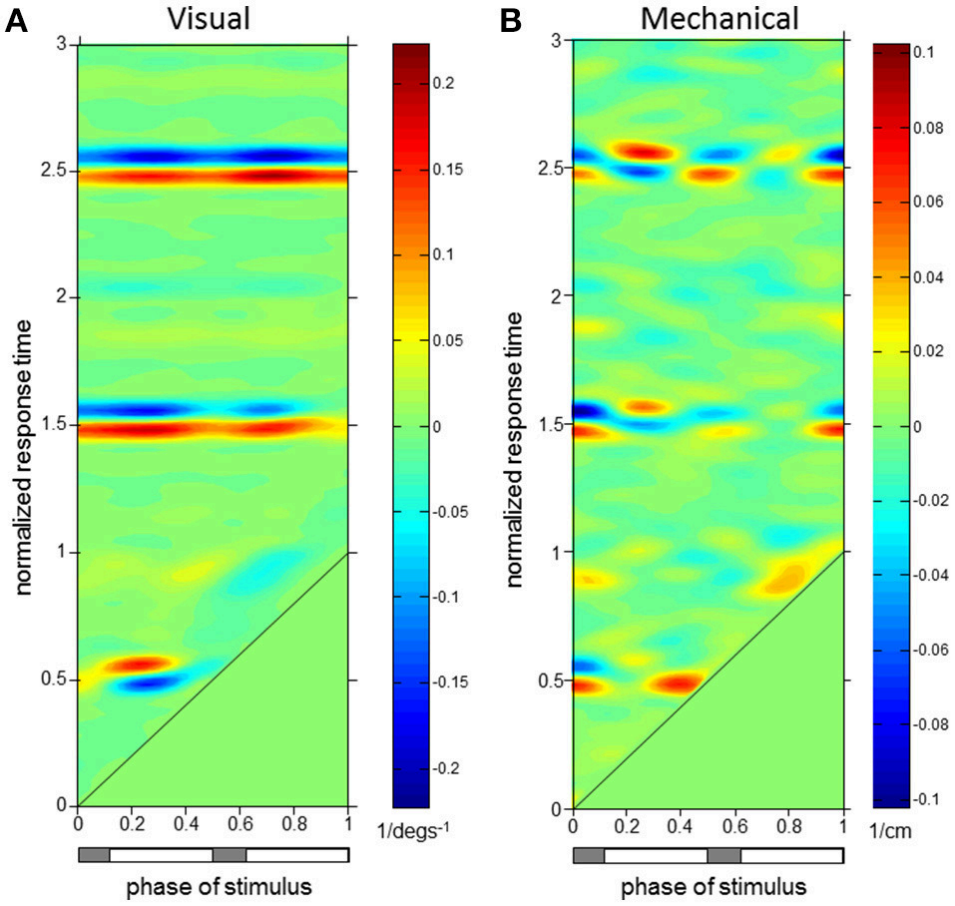
**Trunk Extensor (EST) ϕIRFs. ϕIRFs from visual scene velocity (A)** and motor displacement **(B)** to erector spinae at T9. Intensity of colors indicate magnitude and direction at the plotted combination of stimulus phase and normalized response time. The diagonal black line is where stimulus phase is equal to the normalized response time, which indicates stimulus onset. The horizontal bar below indicates either double limb or single limb support phases in gray and white, respectively.

**Figure 7:**
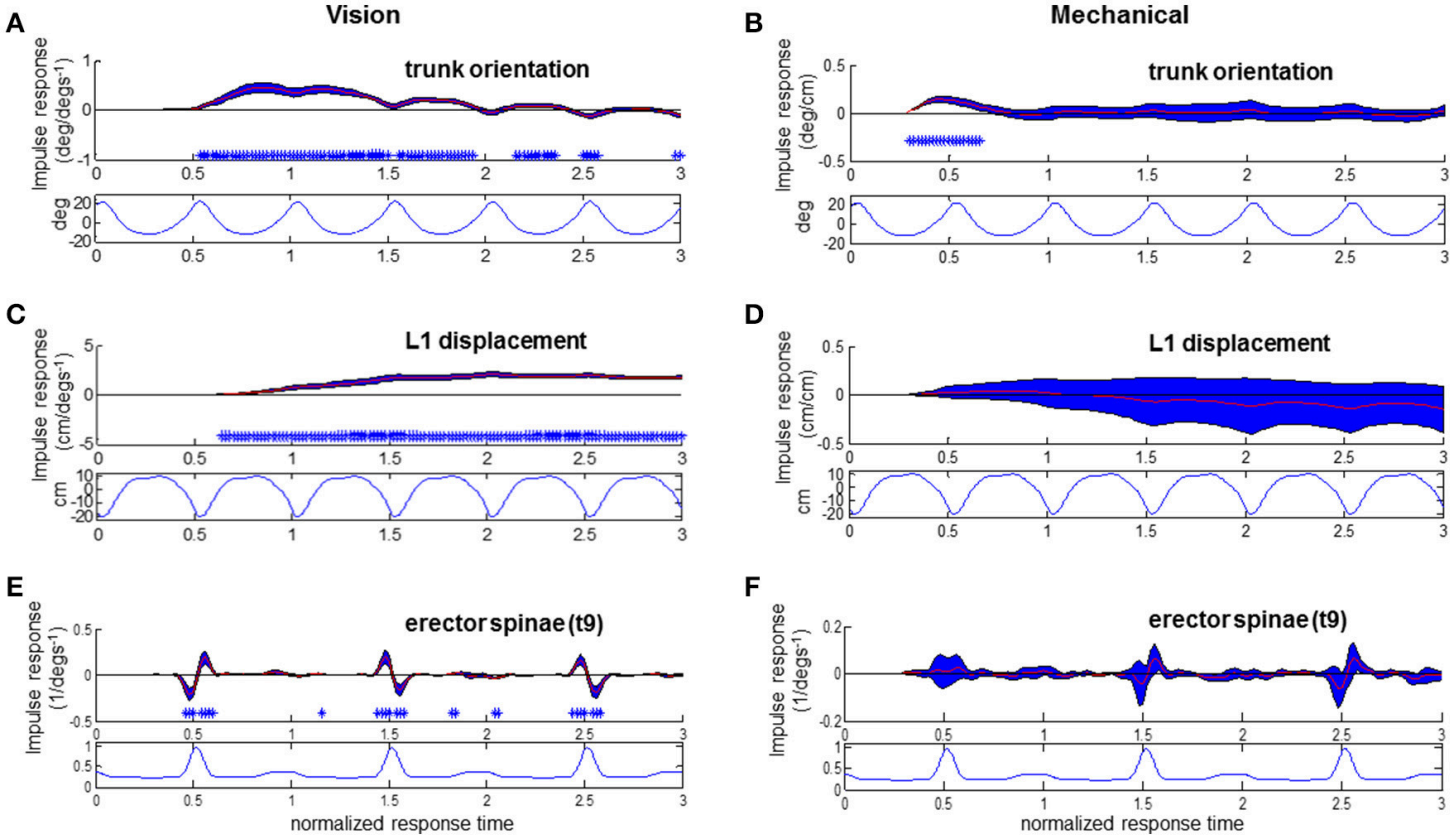
**Responses to visual and mechanical perturbation at 28% stimulus phase**. Impulse response functions of trunk orientation **(A,B)**, L1 AP displacement **(C,D)** and normalized erector spinae activations **(E,F)** to motor position and visual scene velocity. Mean waveforms are plotted below impulse response functions. Shaded blue error bars represent confidence intervals at increment of normalized response time. Asterisks at base of subplots indicate significant difference from zero at increment of normalized response time, corrected for the multiple comparisons made within the stimulus phase (*p* < 0.05).

**Figure 8:**
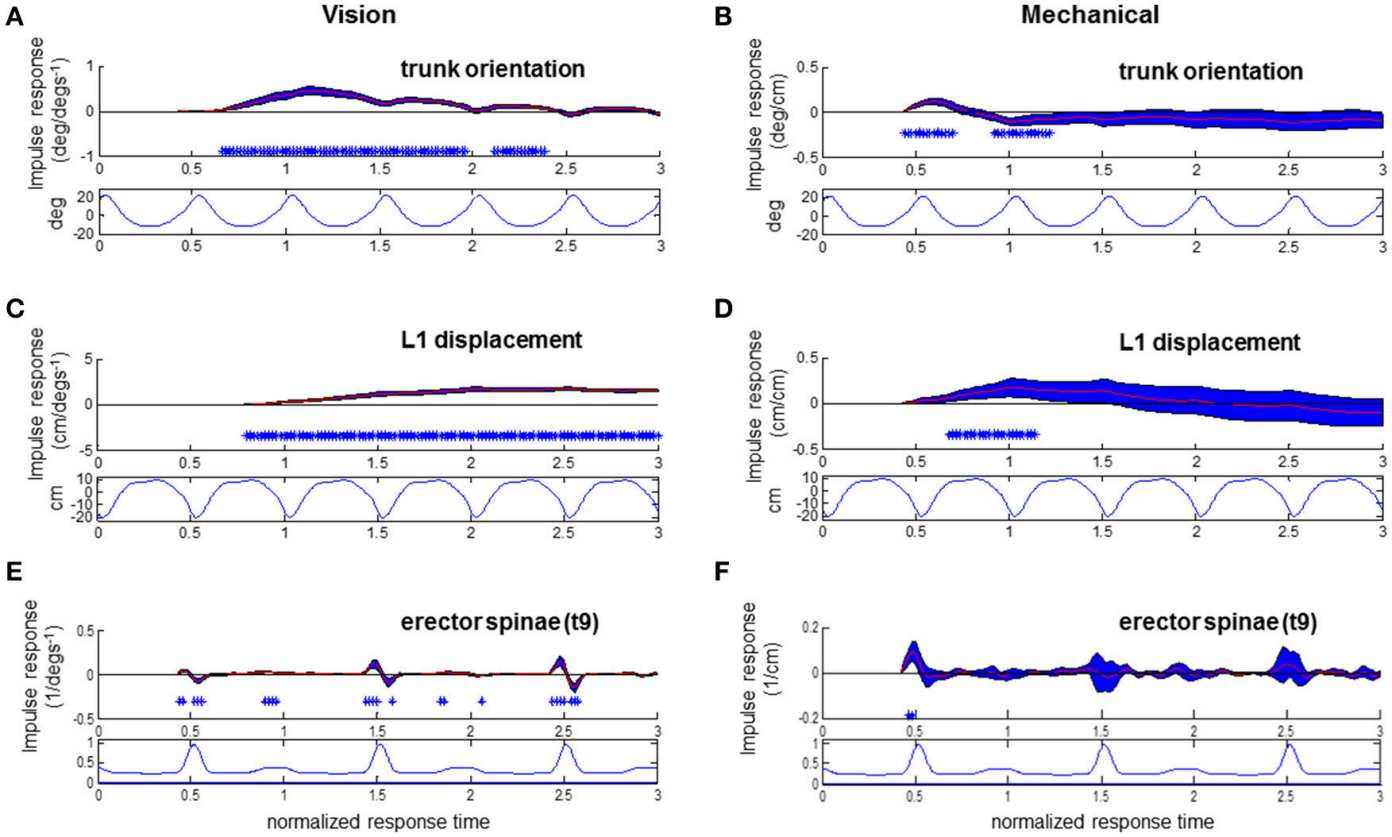
**Responses to visual and mechanical perturbation at 42% stimulus phase**. Impulse response functions of trunk orientation **(A,B)**, L1 AP displacement **(C,D)** and normalized erector spinae activations **(E,F)** to motor position and visual scene velocity. Mean waveforms are plotted below impulse response functions. Shaded blue error bars represent confidence intervals at increment of normalized response time. Asterisks at base of subplots indicate significant difference from zero at increment of normalized response time, corrected for the multiple comparisons made within the stimulus phase (*p* < 0.05).

Steps 1–4 were computed on a trial-by-trial basis with averages of PSDs and CSDs taken across trials for each subject for completion of the HTF analysis and to compute the full ϕIRFs in step 6. Full ϕIRFs are shown in Figures 4–6, with vertical slices in Figures 7, 8 showing the impulse response function at specific stimulus phases. Full ϕIRFs defined above are now termed ϕIRFs in the following text.

The ϕIRF for mechanical perturbations is a response to an impulse in motor position while the ϕIRF for visual perturbations is a response to an impulse in visual scene velocity, which is equivalent to the response to a step in visual-scene position. A positive impulse response (i.e., a positive response) indicates that the variable's response is in the same direction as the perturbation and a negative impulse response (i.e., a negative response) indicates that the variable's response is in the opposite direction as the perturbation.

#### Statistics

Statistical tests of the ϕIRFs of all response variables were performed at each stimulus phase. For illustration, confidence intervals computed based upon the sample mean using the Matlab function “normfit” are plotted in Figures 7, 8. Permutation tests (1000, Manly, 1997) based on the t-statistic (null hypothesis mean = 0) at all normalized response times up to three cycles post stimulus onset were tested simultaneously and family-wise error rate (FWER) was controlled at each stimulus phase for each response variable. The tmax method (Blair and Karniski, 1993) was used to adjust the *p*-value for each value at values of normalized response time within each stimulus phase (alpha = 0.05). These tests were performed in functions written by Groppe (Groppe et al., 2011). These tests are non-parametric and suited for this study as FWER control is strong compared to other methods (e.g., cluster-based permutation testing, false discovery rate) allowing determination of reliable effects in the ϕIRFs (Groppe et al., 2011).

## Results

Phase-dependent impulse response functions (ϕIRFs) presented in Figure 4 show responses of trunk orientation to mechanical perturbations (input is motor position) and visual perturbations (input is visual-scene velocity). Although ϕIRFs were computed based on responses to continuous perturbations, they predict the response to a small brief perturbation applied at any phase of the gait cycle and, by extension, the response to any small transient perturbation (Equation 1). Color represents impulse response value and responses have been plotted as a function of both stimulus phase and normalized response time, the time at which the response is measured in units of cycles. A ϕIRF value is the amount of change in the response variable divided by the integral of the perturbation. For the visual perturbation, a small brief perturbation in visual-scene velocity is equivalent to a small step in visual scene position, so the ϕIRF value is the change in the response variable divided by the change in visual-scene position.

Normalized response time is time divided by the mean gait cycle period $\bar{T}$ of the given trial (1.04 ± 0.05 s, mean ± s.d. across subjects). Doing so allowed a gait cycle-based representation of responses when the perturbation occurred (stimulus phase) and when the response did or did not occur (normalized response time). For example, if $\bar{T}$ = 1.1 s, a heel strike occurs at time 0 s, a perturbation is applied at time 0.55 s, and the response is measured at time 1.1 s, then stimulus phase is 0.5 and normalized response time is 1. For readability, we describe responses to positive perturbations: a brief increase in visual scene velocity or a brief transient forward movement of the motor. From the definition of a ϕIRF (Equation 1), it follows that a negative perturbation would produce the opposite response.

For both perturbations, initial trunk orientation responses were observed as forward rotations at all stimulus phases, as indicated by the diagonal red band observed in both Figures 4A,B which notes positive responses across phases. Put simply, the trunk rotates forward in response to either a brief increase in visual scene velocity or a brief transient forward movement of the motor.

The red band in both figures is approximately parallel to the black line noting stimulus onset, indicating that onset of the response occurs with similar time delay across all phases in which the stimulus occurs. On average across stimulus phases, peaks of the initial forward trunk rotation to vision observed in Figure 4A occur at 0.68 ± 0.06 (mean ± s.d.) cycles (normalized response time) after stimulus onset. As indicated by the black diagonal line in Figure 4, stimulus onset shifts based on stimulus phase, which means that these peak responses are occurring on average 0.68 cycles (normalized response time) in Figure 4A from the black diagonal line at each stimulus phase with small variability across stimulus phases. These initial peaks observed as darker red regions in Figure 4A have an average peak response value of 0.40 ± 0.05 deg/(degs^−1^), indicating a consistent response across stimulus phases. Figure 4B shows that initial peaks in forward trunk rotation to the motor displacement occur with comparatively shorter latency than responses to vision, with average peak responses occurring at 0.17 ± 0.01 cycles (normalized response time), or 0.18 ± 0.01 s, after stimulus onset. These initial peaks in Figure 4B have average peak response value of 0.11 ± 0.02 deg/cm. Interestingly, vertical blue bands indicating a backward trunk rotation to the mechanical perturbation are observed at four stimulus phase ranges in Figure 4B. However, these negative responses are significant (*p* < 0.05 with FWER control, see Methods) only when stimuli are presented at 0.38–0.46 and 0.88–0.96 (“phase of stimulus”) of the gait cycle, which correspond to single limb support phases.

As observed in Figure 5, initial forward responses were also observed in L1 displacement responses to both visual and mechanical perturbations. Forward L1 displacement responses due to visual scene velocity occurred at all stimulus phases and persisted through the 3rd gait cycle of normalized response time. On average across stimulus phases, peaks of the forward L1 displacement due to vision observed in Figure 5A occur at 1.89 ± 0.14 s.d. cycles (normalized response time), or 1.97 ± 0.15 s, after stimulus onset. These initial peaks observed as darker red regions in Figure 5A have an average peak response value of 1.80 ± 0.17 cm/(degs^−1^). Initial, forward displacements due to changes in motor position, on the other hand, were not consistently observed across stimulus phases as seen in Figure 5B. When tested at each stimulus phase, significant responses were observed before and after heel strike at 0–0.22, 0.40–0.68, and 0.96–1 ranges of stimulus phase. Since phase is a circular variable, these values correspond to two ranges of stimulus phase which differ by roughly half a cycle: 0.40–0.68 and 0.96–1.22. Within these ranges, mean peak of the positive response occurred at 0.87 ± 0.30 cycles (normalized response time), or 0.90 ± 0.31 s, after stimulus onset and had average peak response value of 0.21 ± 0.05 cm/cm. Although backward L1 displacements due to changes in motor position were observed in Figure 5B, these were not significant when tested (with FWER control) at each stimulus phase.

Figure 6 demonstrates that erector spinae (EST) responses were dependent on both phase of stimulus and normalized response time for both perturbations. A typical pattern of response in EST to increased visual scene motion is an initial decrease in activation within a cycle after perturbation which is observed as the blue band parallel to the stimulus onset line in Figure 6A. These initial responses are then followed by increased (red) to decreased (blue) bands of activation following at 1.5 and 2.5 normalized response time. This pattern of responses was found to be significant (*p* < 0.05 with FWER control) at the majority of stimulus phases (0.16–0.48 and 0.56–0.82). Also clear from Figure 6A, increased activation does occur after the initial decrease in activation, which was found to be significant at a subset of these stimulus phases (0.16–0.34, 0.76–0.82). Figure 6B shows a comparatively less organized response to the mechanical perturbation, with few of these responses actually being significant. In all, increased activation of EST to the mechanical perturbation was observed in a limited range of stimulus phases including 0.42–0.48, 0.82–0.84, and 0.90–0.92. On average across these stimulus phases, significant responses were observed 0.04 ± 0.02 s.d. cycles (normalized response time), or 0.04 ± 0.02 s, after stimulus onset, and are seen as the red regions which run parallel to stimulus onset in Figure 6B.

To investigate the relationship of the kinematics and muscular activity where significant responses were observed, we focus on specific stimulus phases of the ϕIRFs in Figures 4–6. In Figure 6, clear responses of EST to either the visual scene velocity, motor position or both are seen at the 0.28 and 0.42 stimulus phases. Figures 7, 8 simultaneously show trunk orientation, body displacement and EST at these specific stimulus phases.

As noted in Figures 7A,C with asterisks, significant trunk orientation responses to the visual perturbation occurred prior to L1 displacement responses. At this stimulus phase of 0.28, forward trunk rotations began at 0.54 normalized response time while forward L1 displacements began at 0.64 normalized response time. In Figure 7E, an initial decreased activation at 0.46 normalized response time is followed by an increased activation at 0.54 response time in the EST muscle. This initial decrease in EST activation when virtual scene motion increases velocity occurs prior to forward rotation of the trunk (trunk flexion). Thus, EST decreases its activation prior to trunk flexion when scene motion increases velocity. For the mechanical perturbation, as seen in Figures 7D,F, there are no significant effects of the mechanical perturbation on L1 displacement or EST at this stimulus phase. However, there is a significant forward rotation of the trunk due to the mechanical perturbation occurring at 0.3–0.66 normalized response time, as observed in Figure 7B and observed previously in Figure 4.

At the stimulus phase of 0.42 shown in Figures 8A,C,E, a decreased activation of EST to visual scene motion occurs from 0.52 to 0.56 normalized response time just prior to the initiation of a forward trunk rotation response at 0.66 response time. Once again, a decrease in EST activation occurs with increased virtual scene motion velocity. Trunk orientation responses were initiated prior to L1 displacement responses at this stimulus phase, and at the majority (44/50 observed) of stimulus phases. The pattern of significant EST response followed by trunk orientation responses and then L1 displacement occurred at 28 of 50 stimulus phases, with the specific stimulus phases eliciting this pattern at 0.24–0.44, 0.56–0.82, and 0.92–0.96 of the gait cycle. In all, the combination of responses illustrated in Figures 7, 8 suggests that the EST muscle typically facilitates the response of trunk orientation to visual scene motion.

Responses of the trunk to the mechanical perturbation shown in Figures 8B,D,F also show perturbation induced deviations in trunk orientation occurring prior to deviations in L1 displacement. Noted with asterisks at the stimulus phase of 0.42 shown in Figure 8B, significant forward trunk rotations are initiated at 0.44 normalized response time while forward L1 displacements are first observed at 0.7 normalized response time. As the motor perturbation will first cause responses observed in kinematics which reflect passive responses of the body to decreased pull of the motor-spring apparatus, sEMG responses to the mechanical perturbation are a critical indicator that an active, neural driven response to the mechanical perturbation has occurred. Significant, increased activations of EST were first observed at 0.46 normalized response time at the 0.42 stimulus phase observed in Figure 8F. This occurs prior to initiation of the downward trend of the trunk response at 0.62 normalized response time. At this stimulus phase, the downward trend in trunk orientation results in a significant backward trunk rotation from 0.92 to 1.2 normalized response time. The positive response of the trunk extensor indicates an increased EST activation when the motor is moved forward. A forward motion of the motor decreases the backward force of pulling at the trunk to cause trunk flexion, which results in an increased activation of EST, a trunk extensor, to initiate trunk extension. Significant increases in EST activation due to change in motor position were also observed at 0.44–0.48, 0.82–0.84, and 0.90–0.92 stimulus phases, and were always observed after an initial trunk flexion and prior to the decrease from peak of the trunk flexion response. In sum, the EST response observed in Figure 8, in addition to that observed at other stimulus phases, indicates an active response which resists the mechanical effects of changing the motor position.

## Discussion

Continuous, probing visual and mechanical perturbations to treadmill walking were used in this study to learn about the neural control of human locomotion. Coupled with the novel use of phase-dependent impulse response functions to describe locomotor responses to perturbations, these continuous perturbations allowed an efficient investigation of walking control throughout phases of the gait cycle. Modifications of both sagittal plane trunk orientation and L1 A-P displacement due to visual scene motion were observed at all phases in which the perturbation was applied (stimulus phase). This phase-dependent methodology, however, revealed that additional modifications in these kinematic response variables due to mechanical perturbations occurred at different stimulus phases. Responses of the trunk musculature occurred in conjunction with responses of trunk orientation kinematics to each perturbation, and reflect an active, neural-driven response for control of trunk orientation occurring prior to modifications initiated for whole-body displacement. These findings suggest that control for the subtask of trunk orientation is enacted prior to control of the subtask of positional maintenance.

### Subtask timing suggests prioritization

Responses in the trunk resulting from both perturbations showed the initiation of an active response for sagittal plane trunk orientation control prior to onset of responses of L1 displacement, which is an indicator of A-P whole body motion on the treadmill. Decreased responses in EST to changing visual scene motion were observed prior to increased responses in trunk orientation, indicating that EST responses facilitated the observed trunk orientation responses to vision. In the case of increased visual scene velocity, the visual system sensed changes in visual scene motion leading to the perception that the trunk was orienting backwards, or extending, and relayed to spinal centers for proximal musculature to decrease activation and promote trunk extension. For the mechanical perturbation at some stimulus phases, an EST response occurs just prior to the trunk orientation's decrease from peak response. In the case of a forward motion of the motor, the mechanical perturbation decreases force applied to the upper trunk to cause an increased trunk flexion. Proprioceptive afferents in trunk musculature relay this change to the spinal cord and higher for an increase in trunk extensor muscle activation for maintaining trunk orientation upright. The combination of these results suggests both an active resistance to the mechanical perturbation and use of visual scene motion information for maintenance of orientation upright which occurs before active use of vision for positional maintenance on the treadmill.

The notion that one function, or subtask, of locomotion can be prioritized over another is certainly not a new idea. An early example observed in cats found that animals will alter their strategy for responding to electrical stimuli placed at the dorsum of their paw in a phase-dependent manner (Forssberg et al., 1975). So-called “reflex reversals” whereby stimuli used during an animal's support phase increase extensor activation and delay a flexor withdrawal show that the animal prioritizes the subtask of upright stability at the expense of completing the withdrawal task. More recently, this prioritization of subtask has been observed in human walking as the lowering strategy for obstacle avoidance has been shown to decrease step length of the perturbed limb on the treadmill with increased speed needed in ensuing recovery steps (Forner Cordero et al., 2003). Thus, subjects delay how they maintain speed on the treadmill in order to avoid hitting the obstacle, indicating a subtask prioritization that is ultimately related to upright postural maintenance.

The prioritization of subtask in such studies and suggested here is in terms of time. Both the trunk toppling over the moving legs and being too forward or backward on the treadmill would have dire consequences for walking. However, responses in trunk orientation to the visual perturbation were observed before responses in whole body position on the treadmill. One interpretation of this result is that maintaining upright orientation (postural control) within locomotion is a greater concern to the nervous system than maintaining position on the treadmill (positional control).

This subtask prioritization was observed solely in terms of time, however, without clear decrement in quality of positional control at the expense of postural control that would further support the claim that postural control is more important than positional control. There are two factors other than importance that may influence the relative timing of postural and positional responses. First, postural adjustments may occur before positional adjustments because the nervous system can act to change trunk orientation at any phase of the gait cycle (for example, by modulating the activity of the erector spinae muscles), whereas the nervous system can only effectively act to change position on the treadmill at certain phases of the gait cycle (for example, by modulating the activiation of plantarflexor muscles during push-off). Second, trunk orientation may respond before whole body position due to the way walking speed is controlled. That is, the initial changes in trunk orientation are anticipatory changes, required to counteract expected trunk movement that would result from a self-induced speed change. This would be in line with the notion of anticipatory postural adjustments (Massion, 1992) suggested to occur prior to expected perturbations to standing posture or the initiation of stepping.

In sum, a temporal ordering of trunk orientation prior to AP displacement suggests the nervous system's prioritization of trunk orientation control over that for altering speed to maintain position on the treadmill. Whether this temporal prioritization of trunk orientation observed during walking is driven by importance of the postural control subtask to the nervous system, biomechanical constraints of the walking behavior, or anticipatory postural adjustment for changing speed is not yet clear. Teasing these alternatives apart will take further experimentation including increased task constraints, such as limiting trunk motion and/or use of a self-paced treadmill that does not require subjects to adjust position on the treadmill.

**Figure 9:**
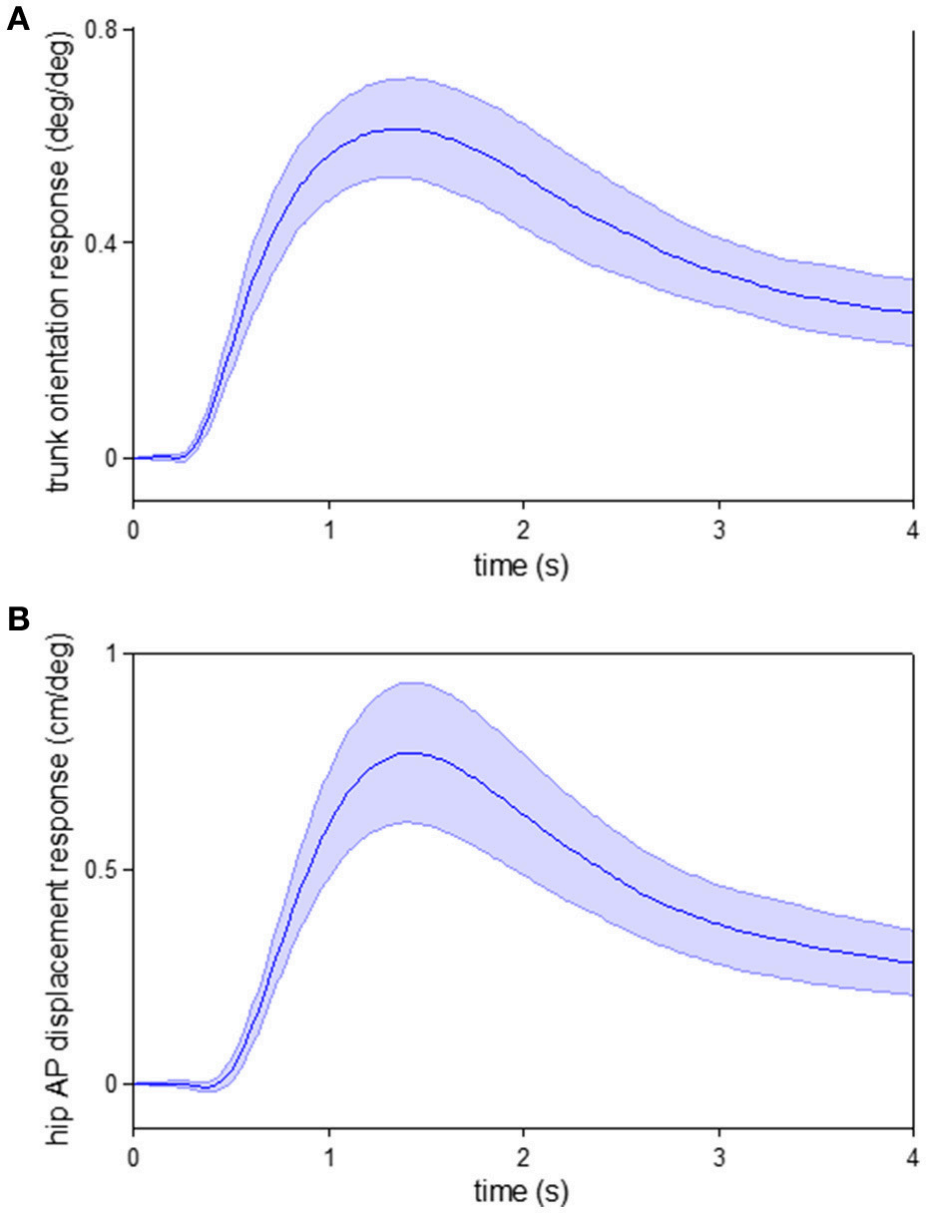
**Responses to visual perturbation during quiet stance**. Impulse response functions of trunk orientation **(A)** and hip AP displacement **(B)** to visual scene velocity. Shaded error bars represent confidence intervals at increment of time. These data were obtained from a previous posture experiment where subjects stood upright (“quiet stance”) in the same visual cave (see Kiemel et al., 2011 for experimental details).

Interestingly, if we apply a similar impulse response function analysis used here on data collected in a previous posture experiment (Kiemel et al., 2011) where subjects stood upright (“quiet stance”) in the same visual cave, we also observe a response of trunk orientation prior to hip AP displacement, a similar indicator of whole body displacement. As seen in Figure 9, when the visual scene rotates forward, the trunk starts to rotate forward before the hip moves forward. Thus, the same temporal ordering of responses occurs in both standing and walking, suggesting an alternative interpretation that the reason for this temporal ordering in walking is not a subtask prioritization during walking, but stems from the general mechanics of interactions between lower- and upper-body motion and how the nervous system takes these interactions into account to more efficiently control movement.

### A phase-dependence for mechanical perturbations

From Figures 4, 5 in combination with the report of significant responses found above, it is clear that active (neurally-driven) responses to the mechanical perturbation occurred in a phase-dependent manner. These phase-dependent active responses to the mechanical perturbation suggest that the nervous system corrects for mechanical disturbances occurring at critical, destabilizing phases in a reactive manner. Winter and colleagues have shown that the proximal musculature (erector spinae and others) activates prior to heel strike to counteract a destabilizing flexion of the head, arms and trunk (HAT) segment due to posterior hip acceleration occurring at heel strike (Winter et al., 1990; Winter, 1995). The moment of force produced by CNS with combined activations of proximal musculature has been deemed the “balancing moment” while the destabilizing force has been deemed the “unbalancing moment” (Winter, 1995). Tang and colleagues have noted that these results by Winter and colleagues (Winter et al., 1990) were found during unperturbed walking, and suggested they reflect a phase-dependent proactive control when walking is not perturbed (Tang et al., 1998). Using perturbations at the support surface they found that proximal muscles of the trunk (rectus abdominus and erector spinae) are not sufficiently modulated during reactions to such stimuli, and do not play a role in active balance responses.

Here we observe a counteracting erector spinae response to a mechanical perturbation which is applied at the trunk, providing a reactive, active balance response. Interestingly, common stimulus phases of both the responses in the erector spinae and the eventual “overshoot” responses in trunk orientation are observed at terminal swing phases in either foot, and these are phases in which Winter's “balancing moment” at the hip is ramping up to its peak to counteract the peak “imbalancing moment” of heel strike. Thus, the reactive response observed here occurs simultaneous with the proactive ramping up of muscular activations for the “balancing moment,” and we can speculate the nervous system's control strategy is to diminish any (internal or external) destabilizing mechanical threats to upright trunk orientation at these critical phases of the gait cycle. In sum, both the site (limb level) of application and gait cycle phase will dictate if the nervous system needs to correct for deviations to a mechanical perturbation during walking.

Clearly, active control in response to the mechanical perturbation must involve sensing the change in trunk orientation at some phase prior to initiating the phase-dependent active response. Phase-dependent stimulation of sensory afferents through perturbations, such as vibration of trunk muscles could likely inform about the role of trunk muscle afferents for these phase-dependent modifications for trunk orientation. Vibration of erector spinae has been successfully performed during walking and has shown that continuous vibration can elicit deviations in walking trajectory (Schmid et al., 2005; Courtine et al., 2007). As phase-dependence in somatosensory inputs of the lower limbs has been well-documented (Duysens et al., 1990; Sinkjær et al., 1996), it is surprising that trunk vibration dependent on gait cycle phase was not tested in those studies (Schmid et al., 2005; Courtine et al., 2007) and has not yet, to our knowledge, been tested in other studies. The question of whether or not somatosensory information regarding trunk motion is available to the nervous system on a phase-dependent basis is an open one.

Somatosensory input may inform that trunk motion has been altered at all phases, yet this input is only used at specific phases. As seen from the impulse responses and mean waveforms in Figure 8, modulation of EST muscle activity to the mechanical perturbation occurs during the phase of the gait cycle that EST is typically most active. The EST activations occurring at early stance observed here counteract the potentially increased “unbalancing moment” at the trunk due to the mechanical perturbation, and prevent inappropriately large flexion of the trunk after heel strike. It is most likely that the observation of active trunk responses to the mechanical perturbation are facilitated by a phase-dependent change in activation, and we suggest that it takes place because the phase of perturbation where the mechanical perturbation occurs is a known preparatory phase for balance adjustments.

### Limitations

This study assumes that walking is the output of a system with a stable limit cycle. We also assume that both intrinsic and external perturbations are small, yielding a local limit cycle (LLC) approximation of the system in which the only nonlinearities are periodic functions of the system's phase (Ermentrout and Kopell, 1984). If the system has a “clock” that prevents phase resetting (for example, walking in sync with a metronome), then the nonlinear functions are periodic functions of time and the system is approximately (LTP) (Möllerstedt and Bernhardsson, 2000). The method used in this study extends the computation of ϕIRFs from LTP systems to LLC systems. However, not all LTP analyses can be extended to LLC systems. For example, for stable linear time varying systems, including LTP systems, one can compute variance accounted for (VAF), the percentage of a system's variance due to its response to a specific perturbation (e.g., MacNeil et al., 1992). This definition of VAF depends on the system's linearity and, therefore, cannot be applied to LLC systems. Phase in a LLC system is a neutrally stable direction, so that phase variability due to perturbations will, in general, grow with time until it is affected by the phase nonlinearities of the LLC approximation (Demir et al., 2000).

### Implications for locomotive control and future directions

A mechanistic extension of the experimental setup used here would be to work within the control theoretic framework of Figure 1 with the long term goal of closed loop system identification (Roth et al., 2014) using the joint input-output (JIO) approach (Katayama, 2005; van der Kooij et al., 2005; Kiemel et al., 2011). Doing so relies on the observation of both kinematic and EMG responses to sensory and mechanical perturbations (Kiemel et al., 2011), and could lead to the non-parametric identification of the musculoskeletal plant and neural feedback for walking, such as that revealed in standing postural control (Kiemel et al., 2008, 2011). This would require a scaling of the analytical tools used for postural control already begun in the HTFs and ϕIRFs used here (Kiemel et al., 2016), and also require considerable advances in experimental methods used for perturbation.

Prior to full identification with use of the JIO, however, one can learn about a system with careful manipulation of experimental conditions. For example, a mechanical perturbation that produces the same kinematic responses but different EMG responses in an experiment with two conditions indicates that properties of the neural feedback change between the two conditions. As we have emphasized trunk orientation control in this experiment, it is expected that an experiment with conditions which require varying needed corrections of trunk orientation, such as use of a backboard or not would elicit changes in EST, and potentially other muscles, contributing to the trunk orientation subtask. We expect that simultaneous mechanical and visual perturbations used during experimental conditions which subjects perform a specific function will inform about how that specific function is controlled during walking. Such experiments offer a novel way to distill out how control differs between subtasks, and offers great promise for distinguishing differences in locomotive control between those with neural deficits and healthy controls.

Our present focus is to work within a system identification framework to investigate the neural control of human walking. However, these tools could be applied to study the neural control of other forms of locomotion approximated as a limit cycle, such as running, cycling, or swimming. Additionally, these techniques are ideal for the study of rhythmic motor behaviors, such as juggling and have already shown promise for application in animal models, such as the isolated lamprey spinal cord (Massarelli et al., 2014, 2015).

## Author contributions

DL, TK, and JJ designed and planned the experiment. DL collected the data. DL and TK analyzed the data. DL and TK wrote the manuscript. DL, TK, and JJ edited and readied the manuscript for submission.

## Funding

Support for this research provided by: NSF grants 0924883 and BCS-1230311 (JJ, TK PIs). Partial funding for open access provided by the UMD Libraries' Open Access Publishing Fund.

### Conflict of interest statement

The authors declare that the research was conducted in the absence of any commercial or financial relationships that could be construed as a potential conflict of interest.
